# Autophagy-lysosome flux failure in diabetic kidney disease: tubular vulnerability and cell-state remodeling

**DOI:** 10.3389/fendo.2026.1957312

**Published:** 2026-09-30

**Authors:** Kaili Hu, Kaini Zhou, Aihua Fei, Cangwu Dong

**Affiliations:** 1 The Second Affiliated Hospital of Anhui University of Chinese Medicine, Hefei, Anhui, China; 2 School of Clinical Medicine, Anhui University of Chinese Medicine, Hefei, Anhui, China

**Keywords:** autophagic flux, autophagy-lysosome axis, diabetic kidney disease, ER-phagy, lipophagy, mitophagy, renal tubule epithelial cells, TFEB

## Abstract

Diabetic kidney disease (DKD) is conventionally framed as a glomerular microvascular complication but may also involve tubular degradative maladaptation, in which tubular cells progressively fail to clear damaged organelles, lipid droplets, and stress-related cargo through the autophagy–lysosome system. Among tubular compartments, proximal tubular epithelial cells depend particularly heavily on mitochondrial metabolism, endocytic trafficking, vesicular transport, and lysosome-supported proteostasis, making them especially vulnerable to diabetic metabolic stress. This review examines whether autophagy–lysosome failure, encompassing both initiation-level and flux-completion defects, provides a candidate link between adaptive tubular stress responses and maladaptive tubular cell-state remodeling. We appraise therapeutic opportunities for restoring degradative capacity, including standard metabolic and hemodynamic therapies, transcription factor EB (TFEB)- and AMP-activated protein kinase (AMPK)-centered interventions, selective autophagy modulation, and mechanism-guided integrative strategies. We propose a testable, stage-dependent reversibility framework, rather than an established DKD staging system: restoration of autophagy–lysosome flux and lysosomal capacity may be most effective before persistent senescence, ferroptotic vulnerability, and fibrosis become difficult to reverse. Accordingly, we distinguish initiation defects from completion defects using flux-competent criteria and identify the stage-dependent reversibility window as the principal conceptual contribution of this review, rather than a validated clinical staging construct.

## Highlights

- Proximal tubular epithelial cells depend heavily on mitochondrial metabolism, endocytic trafficking, vesicular transport, and lysosome-supported proteostasis.
- In diabetic kidney disease, autophagic impairment may reflect reduced initiation, defective flux completion, or both; their relative contributions remain unresolved and require dynamic flux assays.
- Lysosomal dysfunction, spanning impaired biogenesis, defective autophagosome-lysosome fusion, and reduced degradative capacity, lies at the core of tubular injury.
- Selective autophagy programs, including mitophagy, lipophagy, and ER-phagy, clear distinct cargo but converge on shared lysosomal machinery. We hypothesize that, when lysosomal capacity is limiting, increasing one cargo stream may compromise clearance of others; direct competition has not been demonstrated in DKD.
- Progressive autophagy-lysosome dysfunction may promote oxidative stress, inflammation, senescent remodeling, ferroptotic vulnerability, and fibrosis, but its relationship with senescence is bidirectional and context-dependent.

## 1 Introduction

Diabetic kidney disease (DKD) has long been regarded as a microvascular complication dominated by glomerular injury, yet this view does not fully account for the progressive nephron loss and functional decline that accompany disease progression. Recent reviews and human molecular studies suggest that renal tubular epithelial cells (RTECs) are not passive downstream victims of glomerular filtration-barrier damage but active participants in renal function loss, tissue remodeling, and disease progression (1–3). Work on tubular injury linked to glucose and lipid metabolism indicates, in particular, that tubular epithelial cell damage may have pathological relevance to the onset and progression of DKD (4, 5).

These observations do not establish that tubular injury precedes glomerular injury or acts as the primary causal driver. We therefore treat tubular injury as an under-recognized co-determinant within a glomerular-tubular feed-forward loop: glomerular barrier failure increases megalin/cubilin-dependent albumin uptake and lysosomal workload in proximal tubular cells (6), whereas injured tubules may amplify inflammation, interstitial fibrosis, and nephron loss (1). Tubular degradative failure may therefore act as an amplifier of DKD progression without being sufficient or universally upstream.

Among nephron cell types, proximal tubular cells face exceptionally high metabolic and degradative demands, because they continuously sustain solute transport, receptor-mediated endocytosis, membrane recycling, and mitochondrial turnover (7). This burden makes these cells critically dependent on intact organelle quality control, vesicular trafficking, and lysosome-supported proteostasis (8, 9). Under diabetic stress, excessive nutrient load, mitochondrial dysfunction, lipid accumulation, oxidative injury, and advanced glycation end products progressively overwhelm this adaptive system, collapsing cellular homeostasis and activating maladaptive stress responses (4, 10–12).

Within this context, we define tubular degradative maladaptation as a candidate framework in which progressive failure of the autophagy-lysosome axis links diabetic metabolic stress to tubular injury and maladaptive tubular cell-state remodeling. Available tubular DKD evidence supports both initiation-level and flux-completion defects, whose relative contributions may vary by disease stage and experimental context (13, 14).

In this review, tubular degradative maladaptation denotes a systems-level mismatch between degradative demand and macroautophagic-lysosomal capacity. Maladaptive tubular cell-state remodeling refers to stress-associated shifts toward inflammatory, senescent, ferroptosis-prone, or profibrotic states without implying developmental lineage commitment. The stage-dependent reversibility window is defined here as a hypothesized functional interval in which autophagy-lysosome dysfunction and maladaptive tubular cell states remain responsive to verified restoration of degradative flux. Its boundaries are functional rather than chronological and should not be equated with conventional clinical stages of DKD. Potential reversibility would require confirmed improvement in cargo clearance together with concordant improvement in tubular injury and cell-state readouts. Detailed candidate criteria and falsification tests are discussed in Section 6.4.

We therefore propose that selective autophagy programs, including mitophagy, lipophagy, and ER-phagy, become progressively insufficient as diabetic stress accumulates. We advance this as a working hypothesis, not an established disease classification. Once these programs can no longer resolve damaged mitochondria, lipid droplets, endoplasmic-reticulum stress, and other stress-related cargo, the unresolved organelle injury may amplify oxidative stress, inflammation, senescence, ferroptotic vulnerability, fibrosis, and progressive nephron remodeling (15–17).

Previous reviews have established the importance of autophagy, mitophagy, lysosomal dysfunction, and lipid-metabolic stress in kidney disease and DKD (4, 7, 8, 13, 14). These topics, however, are usually treated as separate pathological modules, and the distinction between autophagy initiation and flux completion is applied inconsistently when tubular DKD evidence is organized.

Building on previous reviews of autophagy and mitophagy in DKD and selective autophagy across kidney diseases (13, 14), this review grades key tubular-DKD studies by flux-assay adequacy and distinguishes human DKD evidence (primarily associative), experimental DKD evidence, supportive non-DKD kidney-injury or fibrosis evidence, and broader cell-biological evidence or proposed or incompletely validated relationships. It further advances two testable propositions: that the benefit of restoring autophagy-lysosome flux and lysosomal capacity may be stage-dependent, and that selective cargo streams may compete for finite lysosomal processing capacity under lysosome-limited conditions. The stage-dependent reversibility window—not flux-completion failure itself—is therefore the principal conceptual contribution of this review. This evidence-stratified framework is summarized in **Table 1** and **Figure 1**.

**Table 1:** Evidence-stratified appraisal of the principal claims in the tubular autophagy-lysosome framework of DKD.

Principal claim	Strongest supporting evidence	What remains unproven	Refs
RTECs are active participants in DKD progression rather than passive downstream victims of glomerular injury.	Human biopsy, single-cell and spatial-transcriptomic studies demonstrate tubular injury and maladaptive tubular states in DKD.	These cross-sectional data do not establish temporal priority or primary causality.	(1–3, 19, 20)
Tubular injury is an under-recognized contributor and potential disease amplifier within a glomerular-tubular feed-forward loop.	Glomerular barrier injury increases albuminuria, megalin/cubilin-dependent proximal tubular uptake and lysosomal workload; injured tubules may amplify inflammation and fibrosis.	The complete feedback loop has not been demonstrated longitudinally, and tubular injury is not established as universally upstream.	(1, 3, 6, 19, 20)
Tubular degradative maladaptation reflects a mismatch between degradative demand and macroautophagic-lysosomal capacity.	Glucose and lipid overload, AGEs, albumin uptake and mitochondrial injury increase tubular degradative demand; lysosomal dysfunction reduces processing capacity.	This remains an integrative mechanistic framework, not a validated clinical classification or quantitatively defined disease state.	(6–12, 21)
Initiation-level and flux-completion defects may coexist in DKD tubules.	Initiation mechanisms include mTORC1-ULK1 and p53-miR-214-ULK1; completion mechanisms involve TFEB, Rab7a, fusion and lysosomal degradation. All three principal completion studies were flux-informative, but only the AGE study used a lysosomal-inhibitor clamp.	Completion failure has not been established as the universal dominant lesion. Direct human tubular flux measurements and independent validation of the SMAD3-TFEB axis remain limited.	(9, 12, 18, 22–26)
Mitophagy, lipophagy and ER-phagy clear distinct cargoes but converge on shared lysosomal machinery and may become constrained by finite lysosomal processing capacity.	Separate DKD studies support dysfunction of each cargo pathway; general selective-autophagy and lysosomal biology support dependence on shared lysosomal machinery.	Direct competition among cargo streams has not been tested in head-to-head DKD experiments. Coordination or competition among the three pathways should not be assumed.	(14, 18, 21, 27–47)
Persistent autophagy-lysosome dysfunction may contribute to stage-dependent and bidirectional tubular cell-state remodeling.	Experimental studies link autophagy dysfunction with inflammation, senescence, ferroptotic susceptibility, G2/M arrest and fibrosis; some rescue studies suggest reversibility.	The proposed reversibility window has not been validated longitudinally. No validated marker panel currently distinguishes potentially reversible dysfunction from stable senescence, ferroptotic vulnerability, or self-sustaining fibrosis. The net effects on ferroptosis and fibrosis may vary by cargo, stage and injury context.	(15, 16, 22, 37, 48–56)

Evidence from STZ and conventional db/db models is limited by direct STZ tubulotoxicity and incomplete reproduction of advanced human DKD (57–62). Human biopsy and omics evidence is predominantly cross-sectional. Evidence is classified as human DKD evidence (primarily associative), experimental DKD evidence, supportive non-DKD kidney-injury or fibrosis evidence, or broader cell-biological evidence or proposed or incompletely validated relationships. DKD, diabetic kidney disease; RTECs, renal tubular epithelial cells; STZ, streptozotocin.

**Figure 1:**
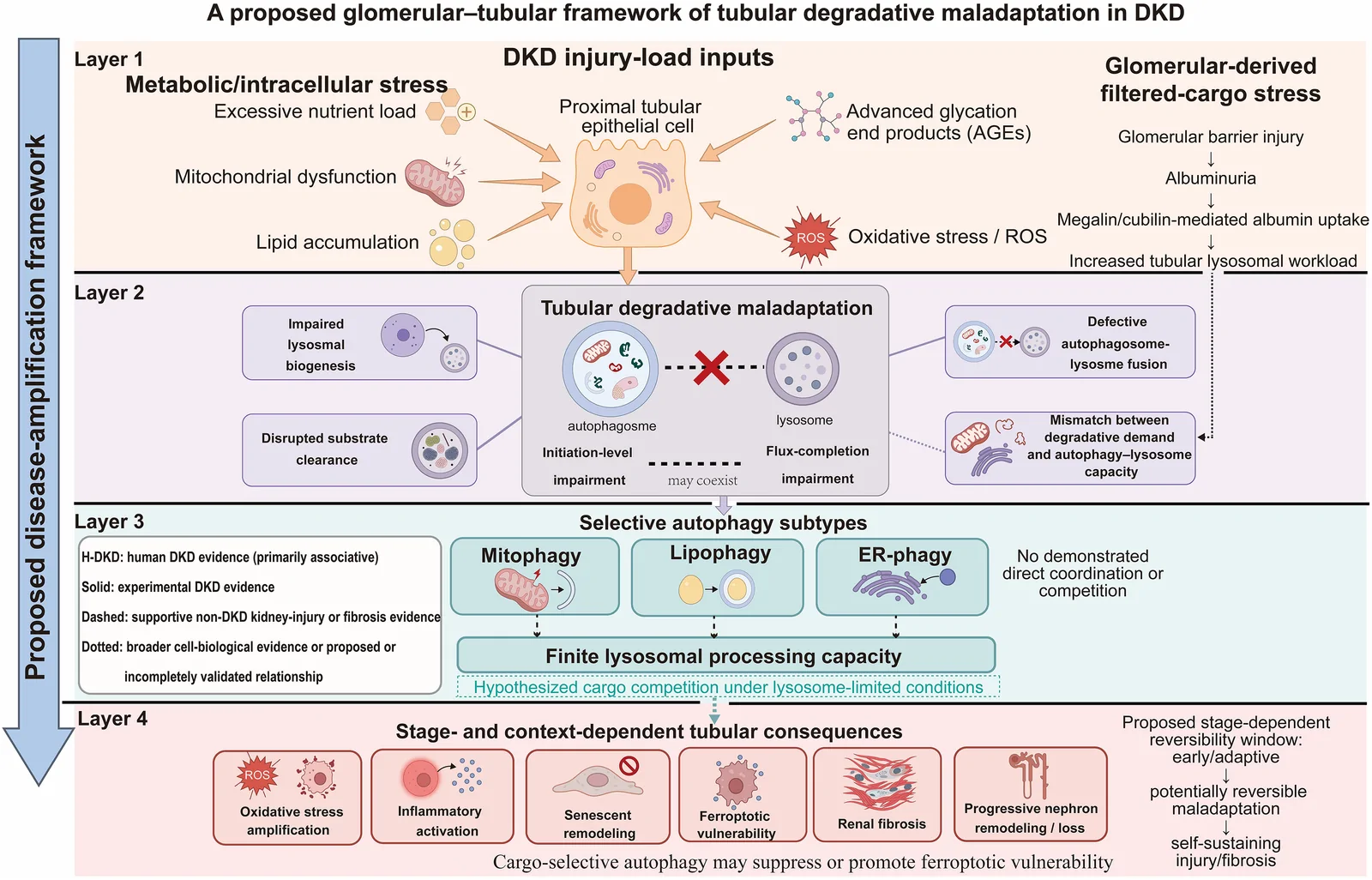
Tubular degradative maladaptation within a proposed glomerular-tubular framework of DKD. Metabolic stress and glomerular-derived albumin load increase tubular degradative demand. When this demand exceeds autophagy-lysosome capacity, initiation- and completion-level defects may coexist and impair selective autophagy. Mitophagy, lipophagy, and ER-phagy converge on finite lysosomal processing capacity, whereas direct coordination or competition among these programs has not been demonstrated in DKD. Persistent maladaptation may contribute to inflammation, ferroptotic vulnerability, fibrosis, and nephron loss. The stage-dependent reversibility window denotes a hypothesized functional interval rather than a validated DKD stage. H-DKD denotes human DKD evidence (primarily associative). Solid, dashed, and dotted connectors denote experimental DKD evidence, supportive non-DKD kidney-injury or fibrosis evidence, and broader cell-biological evidence or proposed or incompletely validated relationships, respectively. Connector style indicates evidence source rather than causal strength.

This narrative review was informed by searches of PubMed conducted through July 2026, without a prespecified lower date limit. Search terms were used in combinations encompassing “diabetic kidney disease” or “diabetic nephropathy”; “renal tubule,” “tubular epithelial cell,” or “proximal tubule”; and “autophagy,” “autophagic flux,” “lysosome,” “mitophagy,” “lipophagy,” “ER-phagy,” “senescence,” “ferroptosis,” or “fibrosis.” Studies were prioritized when they examined human DKD tissue, experimental DKD models, or tubular cell models exposed to diabetes-related stress and reported autophagy-, lysosome-, or cargo-selective-autophagy-related readouts.

Evidence was classified as (i) human DKD evidence (primarily associative); (ii) experimental DKD evidence; (iii) supportive non-DKD kidney-injury or fibrosis evidence; or (iv) broader cell-biological evidence or proposed or incompletely validated relationships. Experimental DKD evidence was considered direct to the tubular DKD framework only when both a diabetic context and a tubular compartment were represented.

Flux-assay adequacy was classified as high when a lysosomal-inhibitor clamp was combined with a tandem reporter or an orthogonal cargo-turnover assay; intermediate when tandem reporters, degradation or fusion assays, or perturbation-rescue experiments were used without a clamp; and low when conclusions relied mainly on steady-state LC3-II, SQSTM1/p62, pathway-associated proteins, colocalization, ultrastructure, or mitochondrial functional readouts. For selective autophagy, cargo-specific lysosomal delivery or turnover was required before completed cargo-selective flux was inferred (18).

Network language here denotes convergence on shared lysosomal machinery, not demonstrated coordination or competition. Under lysosome-limited conditions, we hypothesize that experimentally increasing one selective pathway may reduce the processing capacity available to another: for example, mitophagy induction is predicted to worsen lipid-droplet or ER-cargo retention unless lysosomal capacity is restored concurrently. Concurrent cargo-specific flux measurements during selective activation or inhibition of each pathway, followed by reciprocal rescue after lysosomal-capacity restoration, would support this model; direct head-to-head or reciprocal-rescue experiments remain unavailable in DKD.

## 2 Physiological significance of the autophagy-lysosome axis in renal tubules

Basal autophagy is indispensable for renal tubular homeostasis (7, 63). Unlike relatively quiescent cell types, proximal tubular epithelial cells perform solute transport, receptor-mediated endocytosis, membrane recycling, and mitochondrial turnover continuously (7, 64). These processes impose high metabolic and degradative demands, and they require sustained clearance of damaged proteins, dysfunctional organelles, lipid droplets, and endocytosed substrates (7, 14, 63, 64). Lysosomal remodeling and repair sustain degradative capacity under both physiological and pathological stress (21). The autophagy-lysosome axis is therefore not merely an inducible stress-response pathway in proximal tubular cells but a constitutive homeostatic system that preserves proteostasis, organelle quality control, and epithelial integrity (7, 14, 21, 63, 64). This dependence leaves proximal tubular cells with a narrow adaptive margin: even modest impairment of autophagic flux can promote proteotoxic stress, mitochondrial dysfunction, the accumulation of undegraded substrates, and maladaptive epithelial responses.

In this review, autophagy refers to macroautophagy and its cargo-selective forms unless otherwise stated, reflecting the concentration of direct evidence in tubular DKD (7). Lysosome-associated membrane protein type 2A (LAMP2A)-dependent chaperone-mediated autophagy, microautophagy, and the ubiquitin-proteasome system are important components of renal proteostasis but lie outside the present scope. Megalin/cubilin-mediated endolysosomal processing of filtered albumin, although not a form of macroautophagy, is included because it directly increases proximal tubular substrate load and lysosomal demand in albuminuric DKD (6). Thus, the framework developed below concerns the macroautophagy-lysosome system rather than the entire cellular degradative network.

Lysosomes sit at the center of this adaptive system. They are best viewed not as terminal compartments for waste disposal but as dynamic signaling and metabolic organelles that govern nutrient sensing, calcium homeostasis, stress adaptation, and transcriptional remodeling. Work centered on transcription factor EB (TFEB), together with broader studies of lysosomal remodeling and repair, indicates that lysosomal biogenesis and functional integrity are closely linked to how well epithelial cells adapt to injury (9, 21, 65). Loss of lysosomal competence in tubular cells may therefore contribute not only to inefficient degradation but also to disruption of the broader adaptive network that normally supports epithelial survival and repair.

Recent findings further link lysosomal metabolism to tubular cell identity. Lysosomal cystine export has been shown to regulate mechanistic target of rapamycin complex 1 (mTORC1) signaling and thereby to participate in kidney epithelial cell-fate specialization (66, 67). In parallel, lysosomal dynamics and metabolic functions vary among proximal tubular segments, which suggests spatial heterogeneity of lysosomal homeostasis along the nephron (68). These observations imply that lysosomal integrity sets not only degradative efficiency but also the adaptive boundaries, differentiation state, and stress tolerance of proximal tubular epithelial cells.

The polarized architecture of proximal tubular epithelial cells deepens their dependence on an intact autophagy-lysosome system. Apical-basolateral polarity, continuous protein retrieval, endocytic recycling, and membrane-system turnover all require coordinated vesicular transport, autophagosome maturation, lysosomal fusion, and substrate degradation, so defects in these late-stage trafficking events may substantially compromise proximal tubular homeostasis. DKD-related work on Sestrin2 and Rab7a supports this concept, showing that impaired late endosomal trafficking, defective autophagosome-lysosome fusion, and reduced multivesicular body degradation are associated with tubular injury and disease progression (22).

## 3 Why renal tubules are particularly vulnerable in DKD

Tubular vulnerability in DKD has a multifactorial basis. Metabolically, proximal tubular cells depend heavily on mitochondrial function, and even mild impairment of mitochondrial quality control may increase oxidative burden, deplete adenosine triphosphate, and activate maladaptive signaling (10, 69). Proteostatically, albuminuria and advanced glycation end products add to the burden of endocytic processing and lysosomal degradation, leaving the degradative system more prone to exhaustion (6, 8, 12). Structurally, epithelial polarity and continuous membrane-system turnover render these cells highly sensitive to abnormalities in vesicular transport and fusion (19, 48). The diabetic milieu therefore does not produce a single isolated lesion; it may compress the adaptive reserve of proximal tubular cells at several levels at once.

Both human and experimental studies support the clinical relevance of this framework (20). Tubular injury and interstitial fibrosis correlate closely with renal functional decline in patients with DKD, whereas studies of tubular glucose and lipid metabolism emphasize that abnormal metabolism is associated with direct tubular epithelial injury, although much of the mechanistic evidence is concentrated in proximal tubular models (1, 4, 5). Mitochondrial metabolic reprogramming, moreover, is not a minor accompanying phenomenon in DKD but a candidate mechanism contributing to oxidative injury, inflammatory responses, apoptosis, and autophagic disorder (70). Organizing DKD pathogenesis around failed tubular stress adaptation may therefore provide greater explanatory value than treating tubular changes merely as late-stage or secondary phenomena.

## 4 Major patterns of autophagy-lysosome axis dysfunction in DKD

Autophagic dysregulation in DKD is often attributed simply to abnormal mTOR or AMPK signaling, but that explanation is incomplete. Aberrant upstream nutrient sensing matters; even so, reported lesions in DKD tubules span both autophagy initiation and downstream flux-completion steps, with their relative contributions varying according to disease context and assay design (7, 13). Diabetic stress is associated with a multilayered functional collapse that involves abnormal substrate handling, impaired vesicle maturation, defective fusion, disrupted lysosomal integrity, and insufficient lysosomal biogenesis.

### 4.1 Distinguishing initiation defects from flux-completion defects

The initiation-defect model deserves equal consideration. mTORC1 activity is increased in diabetic proximal tubular cells (23). At the lysosomal surface, the canonical Ragulator-Rag machinery recruits mTORC1 in response to amino-acid sufficiency, enabling mTORC1 to suppress Unc-51-like autophagy activating kinase 1 (ULK1)-dependent autophagy initiation (24, 25). Conversely, reduced AMPK signaling may further diminish ULK1 activation, although this relationship is supported mainly by broader cell-biological evidence rather than direct tubular DKD experiments (25). The p53-microRNA-214-ULK1 axis provides an additional DKD-specific initiation-level mechanism (26). A predominantly initiation-limited state predicts reduced autophagosome formation, whereas a predominantly completion-limited state predicts preserved or increased formation but reduced autolysosome formation, prolonged cargo half-life, or impaired lysosomal degradation. Because the two defects may coexist, they should be distinguished by concurrent measurement of formation and clearance, ideally combining a tandem mCherry-GFP-LC3 reporter with a lysosomal-inhibitor clamp and an orthogonal cargo-turnover assay (18).

Flux-assay adequacy limits the central claim. Of the three principal DKD studies supporting the completion-defect model, all were flux-informative, but only one was pharmacologically clamped. The advanced glycation end-product study used a tandem reporter with bafilomycin, leupeptin, or chloroquine (high adequacy) (12), whereas the SMAD3-TFEB and Sestrin2-Rab7a studies used tandem reporters with degradation, fusion, or rescue assays but no clamp (intermediate adequacy) (9, 22). Because much of the wider literature relies on steady-state LC3-II or SQSTM1/p62, flux-completion failure should be regarded as a testable, context-dependent hypothesis rather than a universal dominant lesion (9, 12, 18, 22).

Disruption of the autophagy-lysosome pathway has been reported in experimental DKD tubular epithelial cell models (9, 12). Autophagic flux is reportedly impaired in DKD, with autophagic substrates accumulating and lysosomal degradation failing (12, 18, 71). Metabolic stress, including glucotoxicity and oxidative stress, has been implicated in lysosomal dysfunction, which may in turn contribute to defective autophagic clearance (12, 72). Collectively, these findings indicate that impaired autophagy in DKD reflects not only reduced activity but also a failure to complete the process.

### 4.2 Lysosomal biogenesis, fusion, and degradative competence

Impaired lysosomal biogenesis is another key lesion. Yang et al. reported that SMAD3 suppresses TFEB-dependent lysosomal biogenesis and thereby contributes to lysosomal depletion and autophagic dysregulation in experimental DKD tubular epithelial cell models. Both pharmacological inhibition and genetic deletion of SMAD3 restored lysosomal biogenesis and improved autophagic flux in these models. This work therefore supplies experimental DKD evidence supporting TFEB as a candidate node connecting profibrotic signaling, lysosomal depletion, and tubular injury (9). However, this axis is supported principally by a single DKD study and lacks independent replication or large-scale validation in human DKD.

Consistent with this view, TFEB-centered interventions restore lysosomal competence in diabetic tubules in animal and cellular models. TFEB activation alleviates tubular epithelial injury in DKD by restoring lysosomal homeostasis, and melatonin may protect lysosomal function partly through TFEB-related pathways (73, 74). Increasing TFEB activity by regulating TFEB acetylation, or by activating the protein phosphatase 2A (PP2A)-TFEB axis with sodium butyrate, may likewise restore autophagy-lysosome function, reduce tubular epithelial cell injury, and alleviate tubular lipid accumulation (75, 76). Impaired lysosomal competence, rather than defective autophagosome formation alone, therefore emerges as a central component of autophagic dysfunction in DKD.

In renal fibrosis models, impaired TFEB-mediated autophagosome-lysosome fusion may promote tubular cell G2/M arrest and fibrotic remodeling through ATPase H+ transporting V0 subunit C (ATP6V0C)- and soluble N-ethylmaleimide-sensitive factor attachment protein receptor (SNARE)-related mechanisms (48). Studies on Sestrin2 suggest that correcting Rab7a-related trafficking defects restores autophagosome-lysosome fusion and promotes multivesicular body degradation (22). Taken together, DKD may involve both initiation-level and flux-completion defects, and neither should be assumed to dominate across all disease contexts, as summarized in **Figure 2**.

**Figure 2:**
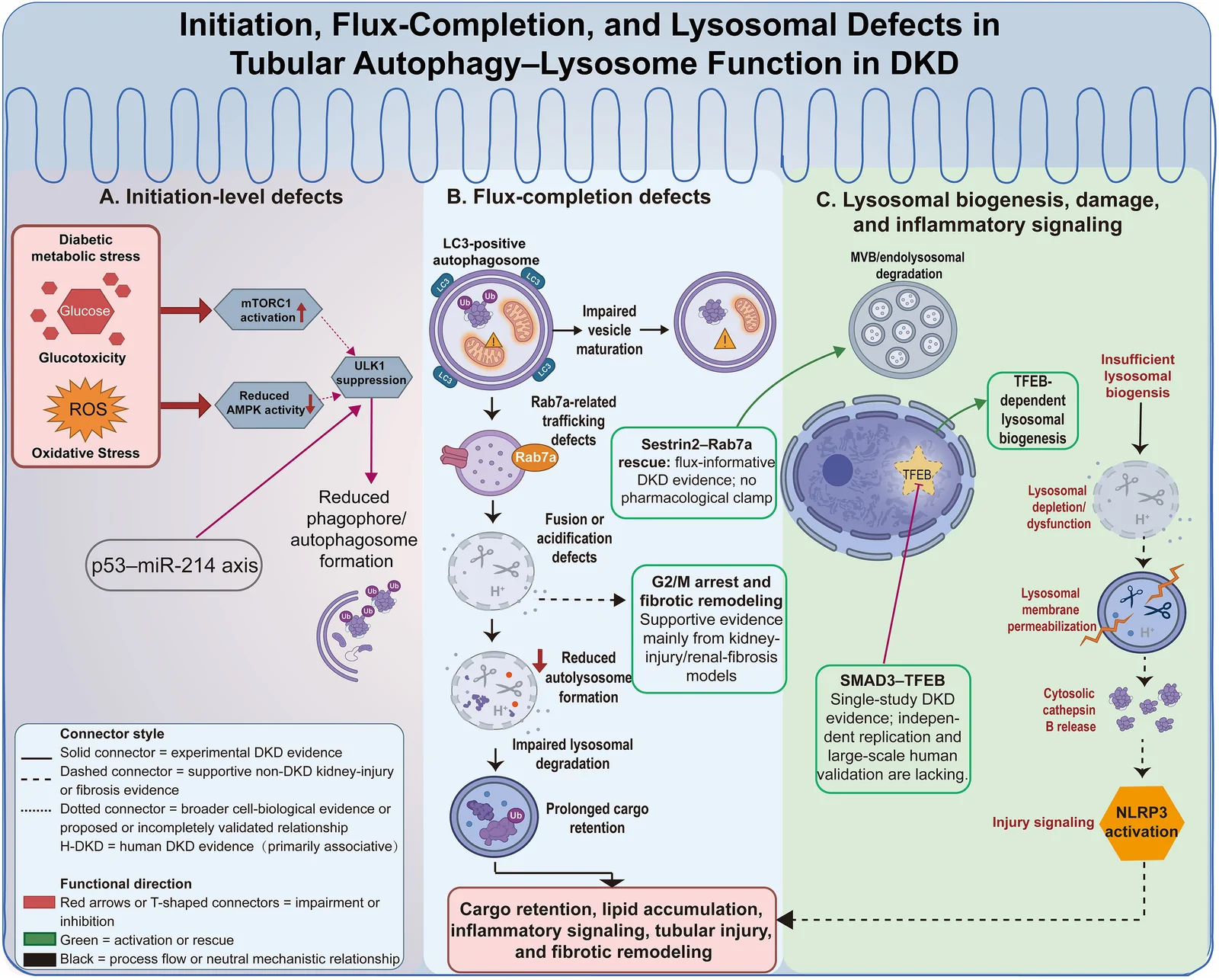
Initiation, flux-completion, and lysosomal defects in tubular autophagy-lysosome function in DKD. Diabetic stress may impair autophagy initiation, autophagosome maturation and trafficking, autophagosome-lysosome fusion, and lysosomal biogenesis or integrity. Initiation-level and flux-completion defects may coexist. SMAD3-mediated suppression of TFEB-dependent lysosomal biogenesis and Rab7a-related trafficking defects represent candidate mechanisms, whereas lysosomal membrane permeabilization may promote cathepsin B release and NLRP3-associated injury signaling. These alterations may converge on cargo retention, lipid accumulation, inflammatory signaling, tubular injury, and fibrotic remodeling. H-DKD denotes human DKD evidence (primarily associative). Solid connectors denote experimental DKD evidence, dashed connectors denote supportive non-DKD kidney-injury or fibrosis evidence, and dotted connectors denote broader cell-biological evidence or proposed or incompletely validated relationships. Connector style indicates evidence source rather than causal strength. Red arrows or T-shaped connectors indicate impairment or inhibition, green arrows indicate activation or rescue, and black arrows indicate process flow or neutral mechanistic relationships.

## 5 Selective autophagy programs in tubular DKD

Of the recognized forms of selective autophagy, mitophagy, which targets damaged mitochondria for degradation, has the strongest experimental evidence base in tubular DKD (7, 13). This evidence is concentrated in experimental DKD models and tubular cell systems, particularly HK-2 proximal tubular cells, whereas available human biopsy findings are primarily associative. Two converging features of proximal tubular biology explain this concentration of evidence: these cells depend heavily on mitochondrial metabolism (10, 77), and diabetic stress is associated with the accumulation of mitochondrial reactive oxygen species, mitochondrial fragmentation, bioenergetic failure, and inflammatory signaling (11, 70, 78). Defective mitophagy may therefore link impaired mitochondrial quality control directly to tubular stress maladaptation.

The AMPK-PINK1-Parkin axis is the best-characterized mitophagy-associated pathway in diabetic tubular models. In HFD/STZ-induced diabetic mice and high-glucose/high-fat-treated HK-2 proximal tubular cells, AMPK activation enhanced PINK1-Parkin-mediated mitophagy and was associated with reduced mitochondrial injury and renal tubulointerstitial fibrosis, whereas AMPK inhibition or PINK1 knockdown weakened these protective effects (27). These reciprocal results support experimental DKD involvement of this pathway but do not establish completed mitophagic flux because lysosome-clamped mitochondrial cargo turnover was not measured. CERS6-derived ceramides were associated with impaired PINK1–Parkin signaling, mitochondrial dysfunction, and renal interstitial fibrosis in DKD models, including HK-2 proximal tubular cells (28). Perturbation–rescue experiments involving CERS6 knockdown and PINK1 interference strengthened this mechanistic link, although the assessment relied mainly on pathway markers and rescue experiments rather than direct measurement of completed mitophagic flux.

Beyond the classical AMPK-PINK1-Parkin pathway, recent studies suggest that mitophagy-associated mechanisms may participate in tubular metabolic adaptation. The ESRRA-ATG5 study combined human DKD biopsy associations with experimental DKD tubular models and linked mitochondrial cargo clearance to arginine metabolism. Its lysosomal-inhibitor-sensitive analyses are flux-informative *in vitro*, although direct *in vivo* mitophagic flux remains incompletely established (29). The XIAP-ULK1 study similarly combined human biopsy findings, diabetic mouse models, tubular cell experiments, and perturbation-rescue analyses, supporting an association among ULK1 degradation, mitophagy-related changes, and carnitine metabolism (30). However, cargo-specific lysosomal turnover was not sufficiently demonstrated to establish completed mitophagic flux. Independently, pharmacological activation of dihydrolipoamide S-acetyltransferase was associated with AMPK-dependent autophagy and mitochondrial protection in HFD/STZ and db/db mice and in HK-2 proximal tubular cells (31). This study supports general autophagy and mitochondrial protection but does not independently establish selective mitophagic flux.

Other regulatory modules widen the mitophagy-associated landscape in DKD. TIPE1 was linked to PHB2 loss, impaired mitochondrial homeostasis, and tubular injury in human DKD tissue, STZ-induced diabetic mice with tubular epithelial cell-specific Tipe1 deletion, and high-glucose-treated HK-2 proximal tubular cells (32). Because the study primarily used PHB2/PINK1 and mitochondrial functional readouts with genetic rescue, it supports pathway involvement rather than completed mitophagic flux. TOMM7 was examined in human DKD tissue, db/db mice, and high-glucose/palmitic-acid-treated HK-2 proximal tubular cells; TOMM7 restoration and Zbtb12-targeted intervention support a TOMM7-PLA2G6-PINK1/Parkin mechanism, whereas the human findings remain associative (33). In STZ-induced DKD mice and high-glucose-treated HK-2 proximal tubular cells, TRPC6-calpain-1 inhibition improved tubular and inflammatory phenotypes together with TEM, PINK1/Parkin, LC3/SQSTM1, and LC3-COX IV colocalization readouts (34); these measurements are mitophagy-associated but do not constitute dynamic mitophagic flux. Finerenone improved mitochondrial and injury phenotypes in HFD/STZ mice and HK-2 proximal tubular cells, but its mitophagy-related evidence was based mainly on steady-state LC3-II, SQSTM1/p62, ATG5, ATG7, BECN1, and mitochondrial functional readouts (35). VDR activation or tubular epithelial cell-specific VDR overexpression restored PINK1 and BNIP3 expression and improved mitochondrial and fibrotic phenotypes in STZ-induced diabetic mice and HK-2 proximal tubular cells (36); however, PINK1/BNIP3 expression and LC3-TOM20 colocalization do not by themselves establish mitophagic flux. Mitophagy thus represents the most extensively studied selective autophagy program in DKD, but the strength of evidence varies across pathways and assays.

These pathways may be provisionally organized hierarchically. Upstream metabolic, transcriptional, and inhibitory regulators—including AMPK, ESRRA, VDR, XIAP, TRPC6-calpain-1, TIPE1, and CERS6-derived ceramides—converge on mitochondrial priming and cargo recognition mediated by TOMM7-PLA2G6, PINK1-Parkin, BNIP3/NIX/FUNDC1, and PHB2. Productive clearance then requires ULK1/ATG5-dependent autophagosome formation and adequate lysosomal flux. This proposed model predicts that ATG5 loss would override ESRRA activation; restoration of ULK1 or PHB2 may bypass XIAP- or TIPE1-mediated inhibition, respectively; and PINK1 deficiency would be expected to blunt the effects of TOMM7 restoration or CERS6 suppression. Lysosomal failure may override all upstream mitophagy-promoting interventions. YME1L-BCL2L13 is better positioned as an additional mitophagy-associated regulatory module rather than solely as a parallel mitochondrial-proteostasis pathway. The supporting study combined human tubular associations, HFD/STZ-induced DKD mice with tubule-targeted YME1L overexpression, and HK-2 proximal tubular cells; however, its evidence was based mainly on LC3-II/COX IV changes, mitochondrial function, BCL2L13 interaction, and perturbation-rescue rather than a cargo-specific dynamic flux assay (37). These proposed relationships are partly supported within individual studies but remain untested by direct epistasis experiments in DKD (27–37, 77).

Mitophagy should not be treated as a monotonic therapeutic target. Available supportive non-DKD kidney-injury evidence indicates that its effects are injury-context dependent: PINK1 deficiency ameliorated cisplatin-induced AKI in one study (38), whereas PINK1-Parkin signaling was protective in ischemia-reperfusion and septic AKI models (39, 40). BNIP3- and FUNDC1-dependent mitophagy was likewise protective in ischemic AKI or ischemic-preconditioning models (41, 42). These studies provide supportive non-DKD kidney-injury evidence and should not be assigned the same evidentiary weight as experimental DKD studies (27–37). The relative importance of PINK1-Parkin, BNIP3, FUNDC1, and NIX/BNIP3L in DKD remains unresolved. Therapeutic studies should therefore quantify mitochondrial mass and function, mitochondrial biogenesis, cargo-specific mitophagic flux, and lysosomal flux together.

Lipophagy is increasingly recognized as a key mechanism linking autophagy impairment to tubular lipotoxicity. Han et al. reported that reduced lipophagy, ectopic lipid deposition, and heightened lipotoxicity coexist in renal tubules from patients with DKD, in *db/db* mice, and in high-glucose-treated HK-2 proximal tubular cells. Mechanistically, AdipoRon increased lipophagy-associated readouts and attenuated tubular injury through AMPK activation, which highlights the AdipoR1/AMPK axis in lipid droplet turnover (43). The human findings are associative. In HK-2 cells, chloroquine-sensitive LC3-II changes in isolated lipid-droplet fractions, organelle colocalization, and pathway perturbation make the study flux-informative, although direct quantitative lipid-droplet turnover was not established *in vivo*.

Lipophagy should not be treated as an isolated lipid-droplet phenomenon but as one facet of a broader metabolic-lysosomal insufficiency. In DKD, disordered glucose and lipid metabolism is associated with oxidative stress, mitochondrial dysfunction, apoptosis, and lipid accumulation in tubular epithelial cells, with much of the mechanistic evidence derived from proximal tubular models. Blocking mPGES-2 genetically or pharmacologically alleviates renal lipotoxicity and slows DKD progression through Rev-Erbα/FABP5 signaling (44). Impaired carnitine-dependent fatty acid oxidation is likewise associated with accelerated kidney injury in experimental and human DKD (45). Together, these findings suggest that lipophagy deficiency belongs within a wider lipid-homeostasis disorder in DKD, in which impaired lipid droplet turnover, defective fatty acid oxidation, mitochondrial metabolic stress, and tubular lipotoxicity may reinforce one another rather than represent a single linear pathway (4, 70).

ER stress has long been studied in DKD, whereas ER-phagy adds mechanistic precision by asking whether damaged ER is efficiently cleared. Yang et al. provided experimental DKD evidence that PACS-2 deficiency may aggravate proximal tubular injury through impaired ER-phagy in STZ-induced diabetic mice with proximal tubule-specific Pacs-2 deletion and in high-glucose-treated HK-2 proximal tubular cells (46). The study implicated the TFEB/FAM134B axis, and used ER-phagosome ultrastructure, LC3-calnexin and LC3-FAM134B colocalization, CKAP4 clearance, and genetic perturbation-rescue analyses. These assays support ER-cargo processing but do not provide a lysosomal-inhibitor clamp or validated dynamic ER-phagy reporter. Human FAM134B transcriptomic findings remain associative. ER-phagy may therefore provide a framework for interpreting persistent ER stress as incomplete resolution. Viewed separately, studies of mitophagy, lipophagy, and ER-phagy suggest that these programs may buffer distinct components of diabetic stress; their coordination and behavior over disease progression remain hypothetical (14, 27, 43, 46).

These pathways should not be assumed to operate as a uniformly coordinated or competitive unit. Under lysosome-limited conditions, we hypothesize that increasing one selective cargo stream may reduce the processing capacity available to others. This hypothesis should be tested by measuring cargo-specific mitophagic, lipophagic, and ER-phagic flux concurrently during selective activation or inhibition of each pathway, with and without lysosomal-capacity restoration and reciprocal-rescue analysis. **Figure 3** organizes these selective macroautophagy programs from upstream regulation and cargo recognition to autophagosome formation and shared lysosomal clearance. **Table 2** summarizes their principal regulatory modules, evidence levels, pathological consequences, and major limitations in tubular DKD.

**Figure 3:**
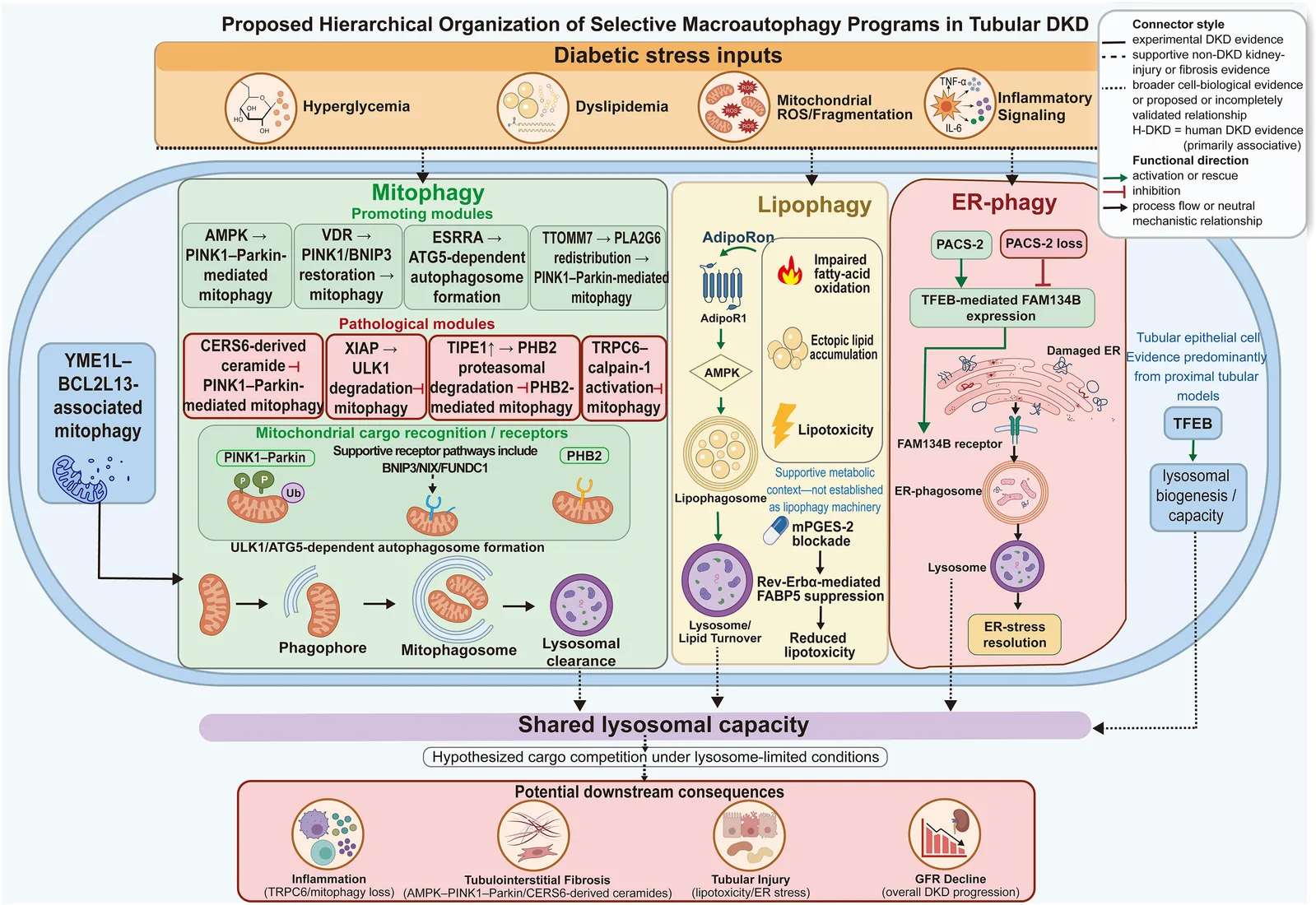
Proposed hierarchical organization of selective macroautophagy programs in tubular DKD. Diabetic stress inputs are depicted as non-exclusive, context-dependent influences on selective autophagy programs. Mitophagy, lipophagy, and ER-phagy are organized from upstream regulation and cargo recognition to autophagosome formation and lysosomal clearance. These programs converge on shared lysosomal capacity; competition for finite lysosomal capacity remains a testable hypothesis rather than a demonstrated mechanism in DKD. Potential downstream consequences may include inflammation, tubular injury, tubulointerstitial fibrosis, and GFR decline. H-DKD denotes human DKD evidence (primarily associative). Solid connectors denote experimental DKD evidence, dashed connectors denote supportive non-DKD kidney-injury or fibrosis evidence, and dotted connectors denote broader cell-biological evidence or proposed or incompletely validated relationships. Green arrows indicate activation or rescue, red T-shaped connectors indicate inhibition, and black arrows indicate process flow or neutral mechanistic relationships. Connector style indicates evidence source rather than causal strength.

**Table 2:** Selective macroautophagy programs in tubular DKD: regulatory hierarchy, evidence sources and major limitations.

Program/cargo	Regulatory hierarchy	Functional interpretation	Evidence source	Major limitations	Refs
Mitophagy—mitochondria	Upstream regulators: AMPK, ESRRA and VDR. Inhibitory modules: CERS6-derived ceramide, XIAP-mediated ULK1 degradation, TIPE1-PHB2 disruption and TRPC6-calpain-1 activation. Recognition/execution: PINK1-Parkin, PHB2, TOMM7-PLA2G6, ULK1 and ATG5; supportive receptor pathways include BNIP3/NIX/FUNDC1. YME1L-BCL2L13 represents an additional mitophagy-associated module.	May maintain mitochondrial quality and support arginine and carnitine metabolism. Benefit may require mitophagic flux to remain balanced with mitochondrial biogenesis and lysosomal capacity.	Human DKD evidence is primarily associative; experimental evidence derives from diabetic animal and tubular cell models, including HK-2 proximal tubular cells. AKI studies involving cisplatin, ischemia-reperfusion, sepsis, or ischemic preconditioning are supportive but non-DKD.	Flux evidence is heterogeneous: most studies rely on pathway markers, colocalization, mitochondrial-function readouts, or rescue experiments; only the ESRRA-ATG5 study provides lysosomal-inhibitor-sensitive evidence *in vitro*. Direct *in vivo* cargo-specific flux, pathway comparison, and epistasis testing are lacking; several studies use STZ-based models.	(27–42)
Lipophagy—lipid droplets	Core pathway: AdipoR1-AMPK-mediated lipophagy followed by lysosomal lipid-droplet clearance. Rev-Erbα/FABP5 signaling and impaired fatty-acid oxidation provide supportive metabolic context but are not established lipophagy-execution pathways.	May limit ectopic lipid deposition and tubular lipotoxicity. Effects on ferroptotic vulnerability may depend on lipid cargo and disease context.	Human DKD evidence is primarily associative; experimental evidence derives mainly from db/db mice and high-glucose-treated HK-2 proximal tubular cells. Kidney-lipotoxicity, lipid-metabolism, and pro-ferroptotic studies provide supportive non-DKD or broader cell-biological evidence.	Direct DKD evidence is concentrated in one principal study. Chloroquine-sensitive LC3-II changes in lipid-droplet fractions and organelle colocalization are flux-informative *in vitro*, but quantitative *in vivo* lipid-droplet turnover remains unestablished. Metabolic studies do not independently demonstrate lipophagic flux.	(43–45, 79–82)
ER-phagy—ER subdomains	PACS-2 and TFEB/FAM134B-associated ER-phagy, followed by ER-phagosome formation and lysosomal clearance.	PACS-2 deficiency is associated with impaired FAM134B-related ER cargo clearance, persistent ER stress and proximal tubular injury.	Human DKD evidence is limited to associative transcriptomic findings. Experimental evidence derives from STZ-induced diabetic mice with proximal-tubule-specific Pacs-2 deletion and high-glucose-treated HK-2 proximal tubular cells; broader ER-stress and ER-phagy studies are supportive.	One principal DKD study used ultrastructure, LC3-calnexin and LC3-FAM134B colocalization, CKAP4 clearance, and genetic rescue, but no lysosomal-inhibitor clamp or validated dynamic ER-phagy reporter. Independent replication and human DKD validation are lacking; STZ-related confounding remains relevant.	(46, 47, 57, 58)
Hypothesized cross-program lysosomal-capacity competition	Mitophagy, lipophagy and ER-phagy converge on shared lysosomal machinery.	We hypothesize that, under lysosome-limited conditions, increasing one cargo stream may constrain the processing of others unless lysosomal capacity is restored concurrently.	Broader cell-biological evidence and framework-level inference; direct competition among cargo streams has not been demonstrated in DKD.	No head-to-head, concurrent cargo-flux, or reciprocal-rescue experiment has directly tested this model in DKD.	(14, 18, 21)

Evidence was classified as human DKD evidence (primarily associative), experimental DKD evidence, supportive non-DKD kidney-injury or fibrosis evidence, or broader cell-biological evidence or proposed or incompletely validated relationships. HK-2 studies were classified as proximal tubular evidence; tubule-targeted in vivo models were not considered proximal-tubule-specific unless explicitly stated. Flux-assay adequacy follows the criteria defined in the Introduction. Proposed hierarchy and cargo competition do not imply established coordination, competition, or epistasis. AKI, acute kidney injury; DKD, diabetic kidney disease; ER, endoplasmic reticulum; STZ, streptozotocin.

Model limitations are critical in a tubular-injury framework. Streptozotocin enters proximal tubular epithelial cells via GLUT2 and can cause direct tubular injury independently of hyperglycemia, confounding both streptozotocin-only and high-fat-diet/streptozotocin studies (57, 58). Conventional streptozotocin and BKS db/db models generally reproduce early-to-intermediate DKD but show limited tubulointerstitial fibrosis and glomerular filtration rate (GFR) decline; advanced db/db phenotypes often require additional susceptibility, such as uninephrectomy (59–61). Consistent with Animal Models of Diabetic Complications Consortium (AMDCC) criteria for progressive DKD—including a greater than 50% decline in GFR and tubulointerstitial fibrosis—no single mouse model fully recapitulates progressive human DKD (62). Mechanistic claims should therefore be cross-validated in non-streptozotocin or advanced-disease models and in human tissue or patient-derived systems.

## 6 Autophagy-lysosome failure and maladaptive tubular cell-state remodeling

Failure of the autophagy-lysosome axis offers a candidate mechanistic link between diabetic metabolic stress and maladaptive tubular cell-state remodeling. Oxidative stress, inflammation, senescence, ferroptosis, and fibrosis in DKD should not be read as isolated downstream events (83, 84). Evidence from experimental DKD models, supportive non-DKD kidney-injury models, and broader cell-biological systems suggests that these processes may represent interconnected responses to unresolved organelle injury and defective degradative flux (85, 86). When damaged mitochondria, lipid-loaded compartments, ER-associated stress signals, and other stress-related cargo go unresolved, tubular epithelial cells may shift progressively from adaptive stress responses toward inflammatory amplification, senescent remodeling, ferroptotic vulnerability, and fibrotic consolidation (87–92).

### 6.1 Oxidative stress and inflammatory amplification

Impaired organelle quality control is a leading source of oxidative stress in diabetic tubules. Defective mitophagy, lipophagy, and ER turnover allow damaged organelles to accumulate, which increases reactive oxygen species and metabolic stress signals (83, 84, 93–95). Excessive reactive oxygen species can in turn damage lysosomes and impair autophagosome maturation, closing a vicious cycle linking organelle injury to defective degradation (93, 95). Studies of renal lipotoxicity and mitochondrial dysfunction support the view that oxidative injury in DKD is closely coupled to autophagy-lysosome failure rather than an isolated metabolic abnormality (77, 79, 96).

Autophagy-lysosome dysfunction also amplifies inflammation. Work on Sestrin2-Rab7a signaling suggests that restoring autophagy-lysosome function reduces deleterious exosome secretion and delays DKD progression, which indicates that impaired degradative flux lets injury spread from initially stressed epithelial cells into the surrounding microenvironment (22). Inflammasome research is consistent with this concept: CD36-dependent mitochondrial reactive oxygen species drive inflammasome activation, and an uncleared injury burden further intensifies proinflammatory signaling (78). Inflammation in DKD may thus be understood as the product of organelle damage, degradative blockade, inflammasome activation, and injury propagation between cells (78, 97–101).

Lysosomal membrane permeabilization (LMP) provides a mechanistic link between tubular substrate overload, inflammation, and cell injury. Excess albumin internalized through megalin/cubilin can induce lysosomal rupture, cytosolic cathepsin B release, and NLRP3 inflammasome activation, whereas urinary-protein overload can also trigger LMP-dependent lysosomal dysfunction and cathepsin-mediated tubular cell-death signaling (102, 103). Cathepsin D may participate in LMP-associated cell death, but direct evidence in DKD tubules remains limited. Thus, LMP may represent a branch point connecting NLRP3-dependent inflammatory amplification with maladaptive tubular injury; this pathway complements mitochondrial reactive-oxygen-species-dependent inflammasome signaling, although the complete sequence remains unconfirmed in human DKD.

### 6.2 Senescence and ferroptotic vulnerability

Persistent autophagy-lysosome failure may contribute to maladaptive tubular cell states by letting damaged organelles and proteins accumulate, thereby favoring senescent remodeling in diabetic tubules (15, 49, 104, 105). This senescence framework matters because senescent tubular cells remain viable yet acquire a senescence-associated secretory phenotype characterized by proinflammatory and profibrotic mediators (49, 106–108). Through that secretory phenotype, these cells may aggravate nephron injury by promoting inflammation, fibroblast activation, fibrotic remodeling, metabolic rigidity, and impaired repair capacity (49, 104, 106–109). More directly, high glucose was associated with increased senescence and impaired autophagic flux in STZ-induced diabetic rats and in HK-2 proximal tubular epithelial cells (15), and CAV1-mediated blockade of CaMKK2/AMPK-dependent autophagy further supports the link between autophagy suppression and tubular senescence (105). Consistently, YME1L-associated enhancement of mitophagy attenuated senescent phenotypes in HFD/STZ-induced DKD mice with tubule-targeted YME1L overexpression and in HK-2 proximal tubular epithelial cells (37), which further supports an association between selective mitochondrial clearance and tubular cell-state maintenance.

The relationship between autophagy and senescence is context-dependent and bidirectional. Loss of basal quality-control autophagy can promote damage accumulation and senescence, whereas autophagy can also facilitate the establishment of some senescent states and support senescence-associated secretory phenotype production through the TOR-autophagy spatial coupling compartment (50, 51). Therapeutic interpretation must therefore distinguish homeostatic cargo clearance from senescence-supporting autophagy and define the disease stage and cellular compartment being targeted (49, 108).

Ferroptosis is a second cell-state outcome linked to degradative failure. Diabetic tubular injury models have identified ferroptotic cell death and ACSL4-dependent lipid peroxidation as major amplifiers of tubular damage (110, 111). Defective autophagy and AMPK inactivation were associated with increased ferroptotic susceptibility in diabetic proximal tubular cells (16). Senescence and ferroptosis are therefore not parallel endpoints lying outside autophagic dysfunction but key manifestations of a prolonged imbalance in the degradative network (16, 111, 112).

Autophagy is not uniformly protective against ferroptosis. NCOA4-mediated ferritinophagy expands the labile-iron pool, whereas lipophagy mobilizes peroxidizable lipid substrates (80–82). Evidence for these cargo-selective pro-ferroptotic pathways comes mainly from non-DKD systems; whether they are dissociable from failure of bulk quality-control autophagy remains unresolved in diabetic tubules. Therapeutic strategies should therefore restore balanced, cargo-appropriate flux rather than maximize autophagy or lipophagy.

### 6.3 Fibrotic remodeling and intercellular propagation

Fibrosis can be read as the tissue-level consolidation of unresolved tubular stress rather than a separate endpoint downstream of injury. Supportive non-DKD kidney-injury or fibrosis studies support a model in which stressed epithelial cells may contribute to fibrotic remodeling through autophagy-dependent secretory programs, TFEB-related pathways, and tubular exosomal cargo (17, 113, 114). Broader kidney-disease reviews reinforce this evidence by linking autophagy dysfunction, lysosomal stress, and maladaptive epithelial repair to tubulointerstitial fibrosis (115), as do DKD-focused studies showing that senescent or stressed tubular cells can reinforce extracellular-matrix deposition, fibroblast activation, and impaired epithelial recovery (49, 106, 107, 116, 117). Fibrosis in DKD therefore likely reflects sustained organelle injury, impaired degradative flux, inflammatory amplification, and failed epithelial recovery acting together rather than loss of autophagy alone.

### 6.4 Reversibility of maladaptive tubular cell states

This framework makes it necessary to distinguish potentially reversible autophagy-lysosome dysfunction from stable or self-sustaining maladaptive tubular cell states. Operationally, a state would be considered potentially reversible only when verified restoration of degradative flux is accompanied by concordant improvement in tubular injury and cell-state readouts. Candidate degradative criteria include dynamic autophagic or cargo-specific flux, substrate clearance, lysosomal acidification and protease activity, lysosomal membrane integrity, TFEB nuclear translocation, and lysosomal biogenesis (9, 12, 18, 22, 102). Candidate state-specific readouts include sustained p16 and p21 expression, SA-β-gal activity, and persistent G2/M arrest for stable senescence; reduced GPX4, increased ACSL4, lipid peroxidation, and labile iron for ferroptotic vulnerability; and persistent YAP/TAZ and TGF-β signaling, extracellular-matrix accumulation, and myofibroblast activation for self-sustaining fibrotic remodeling (15, 16, 48, 49, 52–54, 104, 105, 110, 111). These readouts should be interpreted as a candidate multiparametric panel rather than a validated clinical staging system. The evidence for this reversibility hypothesis remains suggestive rather than definitive: it rests on associative human biopsy evidence, experimental rescue studies performed at defined disease time points, and inference from broader senescence, ferroptosis, and fibrosis biology (9, 15, 16, 20, 22, 37, 49, 104, 106). G2/M arrest is a potentially reversible checkpoint rather than a terminal state, although persistent arrest may contribute to profibrotic signaling (48, 52). Whether autophagy-lysosome restoration retains efficacy once tubular cells develop stable senescence or ferroptotic vulnerability, or once self-sustaining fibrotic remodeling is established, has not been tested directly in longitudinal human DKD cohorts. Longitudinal studies should prespecify early and late intervention time points, verify target engagement with flux-competent assays, and assess tubular injury, cell-state, and fibrotic outcomes. Rescue confined to an earlier intervention point would support a stage-dependent window; equivalent rescue at later stages would argue against a narrow window; and failure to improve tubular outcomes despite verified restoration of degradative flux would challenge the proposed causal framework. We therefore present the reversibility window as a testable framework for time-resolved multiomics and intervention-timing studies, not as an established clinical staging principle.

Matrix stiffening may narrow the reversibility window by sustaining YAP/TAZ-dependent epithelial plasticity and profibrotic signaling, whereas altered autophagic flux may affect YAP turnover and repair capacity. This proposed feedback loop may convert reversible tubular stress into self-sustaining fibrosis but remains untested in human DKD (53, 54, 69, 118).

No validated biomarker currently measures autophagic flux in human DKD *in vivo*. Candidate proxies include urinary extracellular-vesicle LC3-II and SQSTM1/p62 with cargo-receptor and nephron-segment markers, multiparametric biopsy panels assessing autophagic and lysosomal status, and ex vivo pulse-chase or tandem-reporter assays in patient-derived organoids (18, 55, 56). Urinary and biopsy measures remain steady-state proxies, whereas organoid assays do not directly measure *in vivo* flux and require methodological standardization. Until prospectively validated, the reversibility window remains a research construct rather than a clinical staging tool.

Autophagy-lysosome failure does not simply coexist with oxidative stress, inflammation, senescence, ferroptosis, and fibrosis. It supplies a candidate mechanistic bridge by which diabetic stress is translated into persistent organelle injury, maladaptive tubular cell-state remodeling, and progressive nephron remodeling.

## 7 Therapeutic implications and translational perspectives

The therapeutic value of the autophagy-lysosome framework lies in distinguishing clinically validated upstream injury-load reduction from candidate restoration of autophagy-lysosome flux and lysosomal capacity. Standard-of-care therapies remain the foundation of DKD management, whereas interventions targeting TFEB, AMPK, lysosomal biogenesis, autophagosome-lysosome fusion, and selective autophagy may offer complementary strategies for restoring tubular stress resilience. **Table 3** organizes these therapeutic opportunities by evidence strength, separating clinical outcome evidence from preclinical autophagy-lysosome mechanism evidence. **Figure 4** presents the corresponding evidence-stratified therapeutic framework, separating clinically validated load-reduction therapies from preclinical autophagy-lysosome restoration strategies without assuming that autophagy mediates randomized-trial benefits.

**Table 3:** Therapeutic strategies targeting tubular stress and the autophagy-lysosome axis in DKD, organized by evidence strength.

Evidence level/strategy	Representative interventions	Therapeutic positioning	Evidence boundary	Refs
A. Clinical outcome evidence: standard-of-care therapy	RAS blockade; SGLT2 inhibitors; GLP-1 receptor agonists; finerenone	Reduces upstream hemodynamic, metabolic, inflammatory and oxidative injury load and may thereby lessen tubular workload.	Kidney and cardiorenal benefits are clinically established. Autophagy-specific mediation has not been demonstrated. SGLT2i-mTORC1/autophagy and finerenone-mitochondrial protection are mechanistic intersections, not proven clinical mediators.	(35, 119–124)
B. Preclinical mechanism: TFEB-centered lysosomal-capacity restoration and fusion restoration	TFEB activation; melatonin; sodium butyrate/PP2A-TFEB modulation; inhibition of TFEB deacetylation; SMAD3 inhibition	Candidate restoration of TFEB activity, lysosomal biogenesis, autophagosome-lysosome fusion and degradative capacity, potentially within a reversible maladaptation window.	Predominantly preclinical. SMAD3-TFEB lacks independent replication; ATP6V0C/SNARE-G2/M evidence is mainly from renal-fibrosis models.	(9, 48, 73–76)
B. Preclinical mechanism: AMPK-centered and context-appropriate selective-autophagy restoration	AMPK agonists; dihydrolipoamide S-acetyltransferase activators; AdipoRon; experimental mitophagy, lipophagy and ER-phagy restoration	Seeks balanced selective-autophagy flux and improved mitochondrial, lipid-droplet and ER homeostasis.	Animal and cellular evidence predominates. Optimal effects may depend on cargo, dose, disease stage, mitochondrial biogenesis and lysosomal capacity; no DKD clinical-outcome validation is available.	(16, 27, 31, 43, 46)
C. Hypothesis-generating mechanism-mapped examples	Cycloastragenol-ERK-TFEB; Huangkui capsule-STING1/PINK1; Tangshenning-Sestrin2/AMPK/PGC-1α	Mechanism-mapped natural-product and traditional Chinese medicine examples; not established adjunct therapies.	Mainly one preclinical study per intervention, with limited replication, component attribution, pharmacokinetic characterization, dose justification and clinical validation.	(125–129)

Clinical outcome evidence does not establish autophagy-specific mediation. Preclinical and hypothesis-generating evidence does not demonstrate clinical efficacy. GLP-1, glucagon-like peptide-1; RAS, renin-angiotensin system; SGLT2, sodium-glucose cotransporter 2.

**Figure 4:**
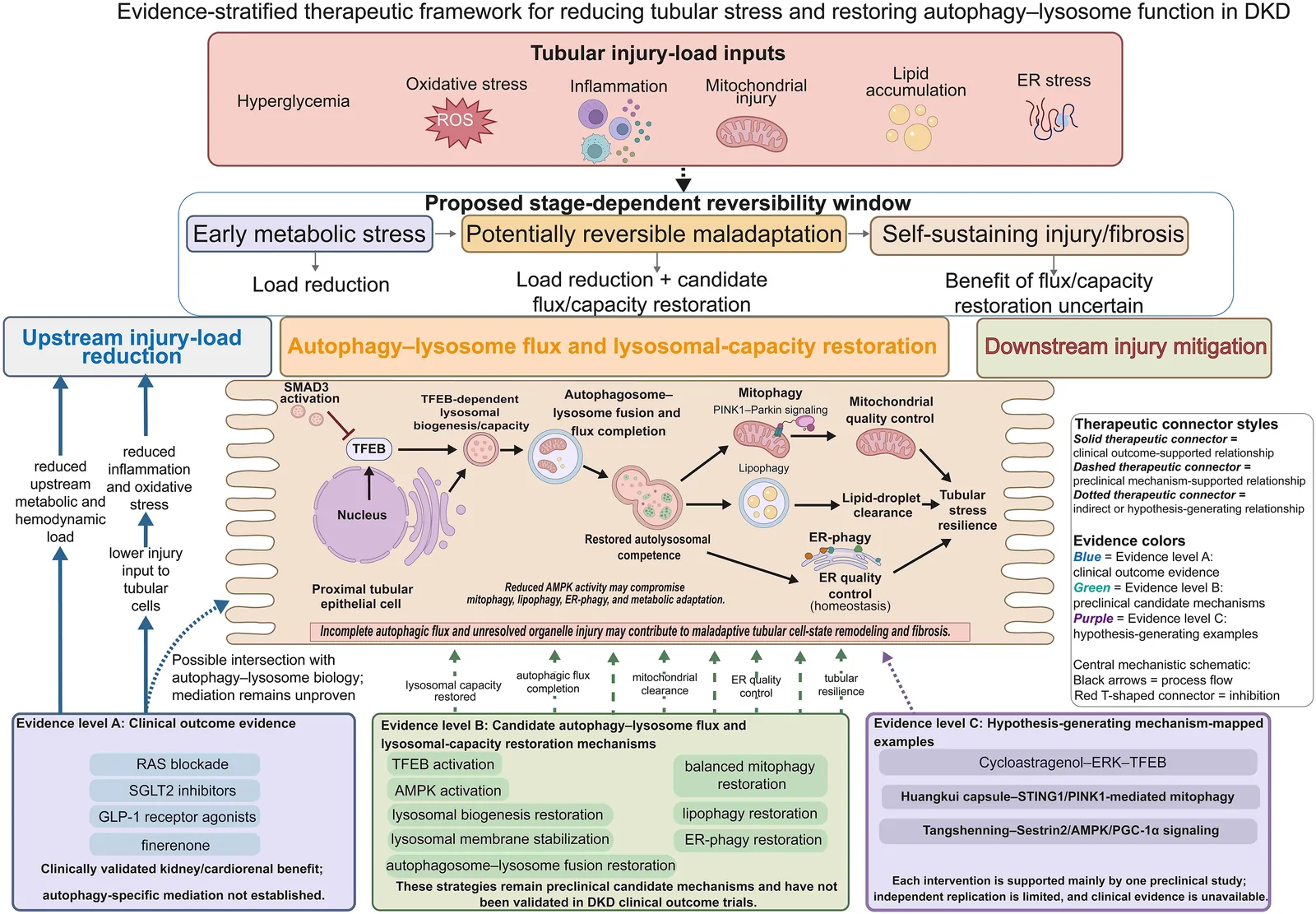
Evidence-stratified therapeutic framework for reducing tubular stress and restoring autophagy-lysosome function in DKD. The upper band presents a proposed stage-dependent reversibility window—a testable functional framework rather than a validated DKD staging system. Tubular injury-load inputs are non-exclusive and may operate across this continuum. Standard-of-care therapies reduce upstream injury load, whereas candidate restoration of autophagy-lysosome flux and lysosomal capacity remains primarily preclinical and may be most relevant during potentially reversible maladaptation. The central schematic depicts SMAD3-mediated suppression of TFEB-dependent lysosomal biogenesis and the potential contribution of restored autolysosomal competence to the completion of mitophagy, lipophagy, and ER-phagy and to tubular stress resilience. Evidence levels A, B, and C denote clinical outcome evidence, preclinical candidate mechanisms, and hypothesis-generating examples, respectively. Blue, green, and purple denote evidence levels A, B, and C, respectively. For therapeutic connectors, solid, dashed, and dotted connectors indicate clinical outcome-supported, preclinical mechanism-supported, and indirect or hypothesis-generating relationships, respectively. Within the central mechanistic schematic, black arrows indicate process flow and red T-shaped connectors indicate inhibition. Clinical benefits of level A therapies do not establish autophagy-specific mediation. This therapeutic evidence scale is distinct from the source-based evidence classification used in **Figures 1**–**3**.

### 7.1 Clinical standard-of-care therapy as upstream injury-load reduction

Any mechanism-guided strategy must rest on established DKD treatment. Kidney Disease: Improving Global Outcomes (KDIGO) recommendations for comprehensive management include renin-angiotensin system blockade, sodium-glucose cotransporter 2 (SGLT2) inhibitors, individualized glucose-lowering therapy, and risk-based use of agents such as glucagon-like peptide-1 receptor agonists and finerenone (119). These therapies chiefly reduce hemodynamic stress, metabolic overload, inflammation, oxidative injury, and cardiorenal risk, and so lighten the upstream burden on tubular epithelial cells.

Importantly, standard therapies may also intersect with autophagy-related mechanisms. SGLT2 inhibitors can mitigate tubular metabolic stress and mTORC1 perturbation, thereby reducing the upstream injury load imposed on proximal tubular cells (120–124). This mechanistic intersection should not be interpreted as evidence that autophagy mediates their established clinical benefits.

### 7.2 Preclinical autophagy-lysosome flux and lysosomal-capacity restoration

In DKD models, SMAD3 suppresses TFEB-dependent lysosomal biogenesis, thereby contributing to lysosomal depletion and autophagic dysregulation (9). Conversely, TFEB activation or TFEB-centered interventions were associated with restored lysosomal homeostasis, reduced tubular epithelial injury, and decreased tubular lipid accumulation under diabetic conditions (73, 75, 76). Melatonin has also been reported to attenuate kidney injury by alleviating lysosomal damage in a mouse DKD model (74). Supportive non-DKD kidney-fibrosis evidence further indicates that TFEB-ATP6V0C/SNARE-related fusion defects may contribute to tubular G2/M arrest and renal fibrosis (48). Therapeutic autophagy activation should therefore be defined not by increased autophagosome formation alone but by whether autophagic flux is completed and lysosomal degradative capacity restored. AMPK-centered and selective autophagy-targeted interventions may improve tubular resilience by restoring balanced mitophagy and supporting lipophagy, ER-phagy, mitochondrial homeostasis, and lipid handling (27, 31, 43, 46). Interpretation of these preclinical findings is additionally constrained by the direct toxicity of STZ to proximal tubular cells and the limited disease progression of conventional STZ and db/db models, as discussed in Section 5. These remain candidate therapeutic strategies rather than validated clinical treatments; whether autophagy-lysosome flux restoration and lysosomal-capacity restoration add benefit beyond standard metabolic and hemodynamic therapy remains unknown.

### 7.3 Mechanism-guided integrative strategies

Traditional Chinese medicine and natural-product interventions are included only as mechanism-guided, hypothesis-generating examples, not as alternatives to standard therapy (125). The cited evidence for cycloastragenol, Huangkui capsule, and Tangshenning formula consists of one preclinical report per intervention, linking them respectively to ERK-TFEB, STING1/PINK1-mediated mitophagy, and Sestrin2/AMPK/PGC-1α signaling (126–128). However, active-component attribution for multicomponent preparations, pharmacokinetics, dose justification, independent replication, and reporting of randomization and blinding remain inadequate; these findings should not be interpreted as evidence of therapeutic efficacy (125, 129).

## 8 Conclusions and future directions

This review frames tubular degradative maladaptation as a candidate mechanism in DKD, through which autophagy-lysosome axis failure may connect diabetic metabolic stress to organelle injury, maladaptive tubular cell-state remodeling, and nephron decline. Four priorities follow: defining the temporal pattern of autophagic flux in human-relevant DKD models and biopsy-based multiomics (130); resolving nephron-segment and cell-state heterogeneity within an integrated glomerular-tubular framework; ranking selective autophagy programs, including mitophagy, lipophagy and ER-phagy, together with sirtuin-linked mitochondrial quality control, by disease stage and cellular context (14, 131); and testing whether restoration of autophagy-lysosome flux and lysosomal capacity adds value beyond standard metabolic and hemodynamic care.

These priorities require clear evidence boundaries. The reversibility hypothesis should be treated as a testable framework rather than a validated DKD staging system, and future studies should determine whether verified restoration of degradative flux produces concordant improvement in senescence, ferroptotic-vulnerability, and fibrotic-remodeling readouts (16, 48, 49). Emerging layers of metabolic control, including mitochondrial protein acetylation and glycolytic lactate metabolism, may further clarify how metabolic remodeling shapes tubular stress and cell-state decisions (5, 132). Overall, this framework may help steer DKD research toward stage-specific, cell-type-specific, and mechanism-guided therapeutic strategies.
